# Patterns of use of symptomatic treatments for Alzheimer's disease dementia (AD)

**DOI:** 10.1186/s12883-023-03447-5

**Published:** 2023-11-09

**Authors:** Julia R. DiBello, Yifei Lu, Jina Swartz, Edward A. Bortnichak, Kai-Li Liaw, Wenjun Zhong, Xinyue Liu

**Affiliations:** 1 Epidemiology, Biostatistics and Research Decision Sciences, Merck & Co., Inc., 770 Sumneytown Pike, West Point, PA 19486 USA; 2 Department of Epidemiology, Gillings School of Global Public Health, University of North Carolina at Chapel Hill, Chapel Hill, NC USA; 3 Exciva EXCIVA GmbH, Hauptstrasse 25, 69117 Heidelberg, Germany

**Keywords:** Alzheimer’s disease, Symptomatic treatment, Treatment patterns, Epidemiology

## Abstract

**Background:**

Symptomatic treatment for Alzheimer's disease (AD) dementia could temporarily slow symptom worsening and improve the quality of life for both AD dementia patients and their caregivers. A comprehensive evaluation of symptomatic treatment patterns using recent data for newly diagnosed AD dementia has not been performed and compared across different countries.

**Methods:**

The drug name, time to the first therapy, duration, discontinuation or switches were described in newly diagnosed AD dementia patients in two databases (a major U.S. health plan [US] and UK-Clinical Practice Research Datalink [CPRD GOLD]). This analysis included patients with newly diagnosed AD dementia in 2018–2019, who initiated symptomatic AD drug therapy, with ≥ 1 year baseline period and ≥ 1 year of follow-up.

**Results:**

Over median follow-ups of 698 and 645 days, 63% and 65% of AD dementia patients used symptomatic treatments, with 34% and 77% newly initiating therapy, constituting analytic samples of 7637 patients in the US database and 4470 patients in the CPRD, respectively. The median time to the first therapy was 14 days for US and 49 days for CPRD; donepezil ranked the as most frequently used (69% vs 61%), followed by memantine (19% vs 28%) in the US database and CPRD, respectively. Median time on first therapy was 213 and 334 days, and 30% and 12% of patients proceeded to a second treatment in the US and CPRD databases, respectively.

**Conclusion:**

Approximately two thirds of newly diagnosed AD dementia patients utilized approved symptomatic treatment. Time on first therapy was relatively short (< 1 year) and the majority did not move to a second therapy, highlighting the need for better adherence and persistence to existing AD symptomatic therapies and the need for additional therapies to alleviate the significant burden of AD dementia.

**Supplementary Information:**

The online version contains supplementary material available at 10.1186/s12883-023-03447-5.

## Background

Alzheimer's disease (AD) accounts for 60–80% of dementia cases and is the sixth leading cause of death in the United States [1]. Most people with AD dementia are 65 and older and exhibit memory loss and other cognitive abilities serious enough to interfere with daily life [2]. Based on the degree of cognitive impairment, AD is often divided into three stages: the preclinical stage, characterized by normal cognitive ability, the prodromal stage, characterized by mild cognitive impairment (MCI), and the dementia stage, with functional impairment [3]. On average, it takes 7 years for an individual with MCI to progress to mild AD, but some individuals may experience a rapid progression which takes significantly less time to develop into AD dementia [4]. In its early stage, memory loss is mild, but with late-stage AD dementia, patients may lose the ability to carry out a conversation and respond to their environment [1]. Caring for AD dementia patients presents unique challenges, including addressing cognitive, behavioral, and communication changes, and providing escalating supervision and personal care as the disease advances, often resulting in heightened emotional stress, financial strain, and employment disruptions for caregivers [1].

Many disease modifying treatments for AD are currently in development. The U.S. Food and Drug Administration (FDA) approved the disease modifying treatments of aducanumab and lecanemab to target brain amyloid for early AD cognitive preservation, [1] yet their safety and efficacy in clinical settings warrant additional investigation [5, 6]. In contrast, more accessible treatments primarily address symptoms, temporarily slowing cognitive deterioration and improving the quality of life for both AD dementia patients and their caregivers [1]. Symptomatic treatment for mild to moderate AD dementia includes cholinesterase inhibitors, such as Aricept® (donepezil), Razadyne® (galantamine) and Exelon® (rivastigmine). These medications have a similar mechanism of action; however, an AD dementia patient may respond better to one drug than another [7]. Gastrointestinal symptoms and sleep disorders are a common adverse effect of cholinesterase inhibitors, typically affecting 5–20% of patients [7]. For moderate to severe AD dementia, licensed symptomatic treatment includes Namenda® (memantine), a glutamate noncompetitive N-methyl-d-aspartate (NMDA) receptor inhibitor [7]. The FDA has also approved Namzaric® for moderate to severe AD dementia, a combination of donepezil and memantine which is considered the most effective symptomatic treatment; this combination has demonstrated fewer gastrointestinal symptoms but an increased occurrence of headaches [8–11].

Current literature reports that a large percentage of patients with AD and related dementias are not on symptomatic anti-dementia treatment [12, 13]. For example, one study among Medicare beneficiaries found that among prevalent patients with AD and related dementias, only 33.3% used an approved anti-dementia drug in the years 2008–2016 [12]. In another study that followed 9812 Medicare beneficiaries with newly diagnosed AD dementia from 2008–2012, only 56.7% initiated symptomatic anti-dementia treatment [13]. In addition, many patients discontinue symptomatic anti-dementia drugs after a period of treatment. For example, within 100 days of starting treatment half of patients from four European healthcare databases, and a quarter of US. Medicare beneficiaries had discontinued treatment [13, 14]. Treatment patterns also vary by regions/countries in terms of drug therapies preferred and treatment duration [13–15]. A comprehensive evaluation of symptomatic treatment patterns using recent data for newly diagnosed AD dementia has not been performed and compared across different countries. It is important to understand current treatment patterns in real-world settings to inform effective disease management.

In this context, we leveraged data from two nationally representative databases, representing countries with varying recommendations for the symptomatic treatment of AD dementia, [16, 17] a major U.S. health plan (US) and the Clinical Practice Research Datalink (CPRD GOLD) in the UK. This study provides an updated description of the treatment patterns of symptomatic AD therapies among patients with newly diagnosed AD dementia in the context of two real-world clinical practice environments.

## Methods

### Data source

The US database is a full dataset consisting of medical, pharmacy and lab claims for Medicare and commercial covered lives linked by a unique patient identification (ID) code/number. The US database is representative of the US privately insured population and the elderly enrolled in Medicare advantage insurance plans [18]. As of 2018, the US health plan included about five million patients with at least one month of enrollment with pharmacy and medical benefits.

CPRD is a governmental not-for-profit observational and interventional research service. CPRD has been collecting primary care data since 1987 providing over 25 years of longitudinal data for public health research. CPRD collects the anonymized patient medical records from general practitioner (GP) practices using two of the three major GP software systems (Vision and EMIS) throughout the UK. As of March 2017 CPRD, held data on > 24 million patients from > 800 GP practices. The CPRD GOLD contains patient-level information on demographics, lifestyle data, clinical diagnoses, prescriptions, and preventive care and has been found to be representative of the UK population with respect to gender, age and ethnic group [19].

### Study population

Consistent with previous studies, ICD-10-CM codes for AD dementia were used to identify eligible patients [4, 13]. The first AD dementia diagnosis (G30.xx) recorded between Jan 1^st^, 2018, and Dec 31^st^ 2019 (identification period) was recognized as the index date [20]. We identified patients who were continuously enrolled from 12 months prior to the index date (baseline period) to at least 12 months after the index date (follow-up period). To meet the eligibility of being newly diagnosed, we required that the patients had no AD dementia diagnosis in the baseline period and had an additional AD dementia diagnosis after the index diagnosis. Individuals were followed until health plan disenrollment, or end of the study period on Dec 31^st^, 2020, whichever came first. Since the primary focus in this descriptive study was to describe treatment initiation patterns in newly diagnosed AD dementia patients, those with symptomatic dementia treatments in the baseline period or without symptomatic dementia treatments documented during the follow-up period were excluded from the study.

In CPRD, similar criteria were applied to select patients with newly diagnosed AD dementia between Jan 1^st^, 2018, and Dec 31^st^, 2019. For CPRD data, patients were only required to have an index AD dementia diagnosis and no previous AD dementia diagnosis in the baseline period. In CPRD, the AD dementia diagnosis was recorded based upon READ codes (Supplementary Table S1).

### AD dementia treatment

In the US database, the first symptomatic AD dementia drug(s) filled on or after the index date and with days’ supply >  = 21 days was counted as the first symptomatic AD dementia therapy. If a patient received two anti-dementia drugs with overlap > 60 days, the patient was considered to be utilizing combination therapy. The start date of the combination therapy was the start date of the second drug, unless the second drug started within 21 days from the start of the first drug. In the latter case, the start date of the combination therapy was the start date of the first drug. Once a patient started a new drug, or dropped a drug from combination therapy, the patient was considered to have moved to their next therapy. Drug discontinuation was defined as no refill of a drug within 56 days after the days’ supply had ended.

In the CPRD database, similar definitions were applied, except that 28 days of supply were imputed for every symptomatic anti-dementia drug prescription due to the large number of missing data values for drug supply days. We imputed a value of 28 for days of supply in the analyses as this was the mode of the distribution of days of supply for symptomatic AD therapies in our patient sample.

### Statistical analysis 

Although we focused primarily on patients with newly diagnosed AD dementia who initiated symptomatic dementia treatment, for comparison with previous studies, we calculated the proportion of patients with newly diagnosed AD dementia who had documented symptomatic dementia treatments during the follow-up period (that is, including patients with baseline medication use prior to the diagnosis as well). Descriptive statistics were applied in this study to summarize the drug name, route (pill or patch), time to start and duration of the treatment. Continuous variables were described by mean/median and standard deviation (SD), and categorical variables were described by percentages. Duration of first symptomatic AD dementia therapy was defined as time from initiation to switch to the second treatment, discontinuation of the first treatment without switching, disenrollment or end of study.

## Results

### US Database

**Fig. 1:**
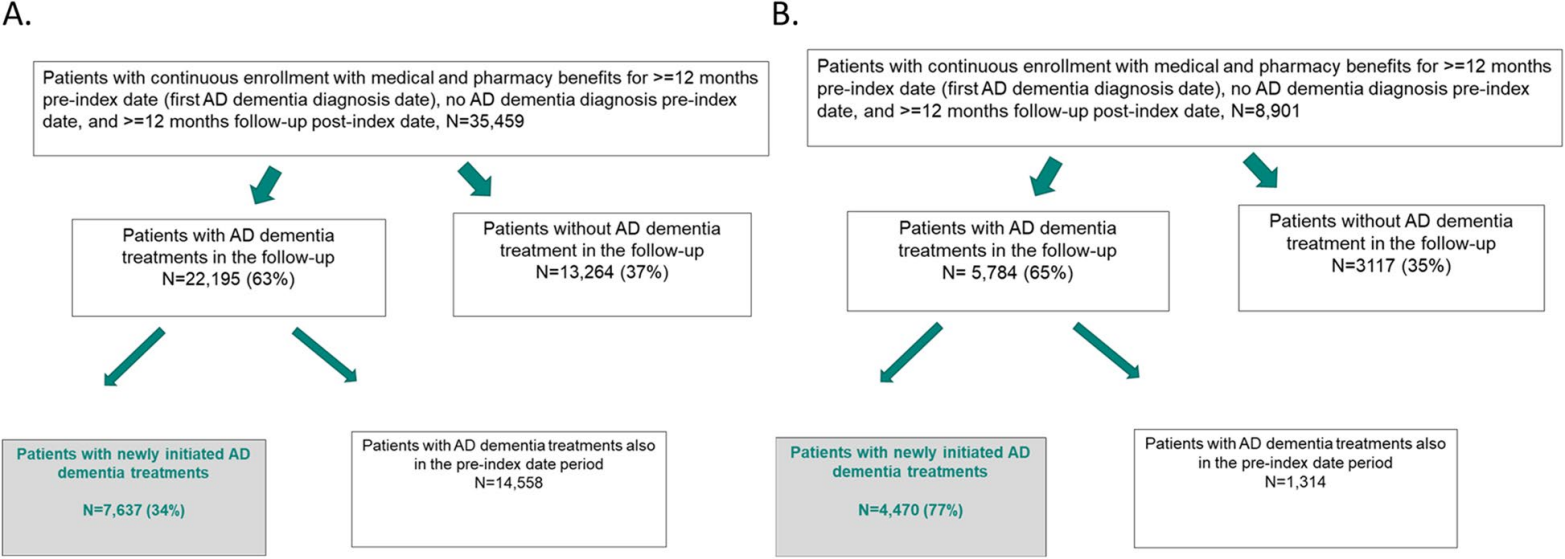
Patient flow from a major U.S. health plan (**A**) and CPRD (**B**) databases

As shown in Fig. 1, of the 35,459 newly diagnosed AD dementia patients, 63% received symptomatic treatments during the follow-up period, of which 34% newly initiated treatment and constituted the final analytic sample. In this sample, there were 7,637 newly diagnosed AD dementia patients without prior treatment history, with 63.2% female and mean age of 82.1 years (SD 11.6 years). Only 1.8% of participants were < 65 years of age. The mean and median follow-up time were 716 and 698 days, respectively. The most frequent co-morbid condition at the index date was diabetes (type 1 and 2 combined), accounting for 32.2% of the patients (Table 1). Overall the characteristics of newly diagnosed untreated AD dementia patients were similar to those initiating treatment; the untreated cohort tended to be older than the treated cohort (Supplementary Table 2).

**Table 1:** Demographic characteristics and co-morbidities in AD patients at index date from a major U.S. health plan database and CPRD

Characteristics	Major U.S. health plan	CPRD
**N**	7,637	4,470
**Age (years)**		
Mean (std)	82.1 (11.6)	81 (7.6)
**Age Group (%)**		
< 65	1.8	2.8
65–74	20.0	15.5
75–84	51.6	47.0
85 +	26.6	34.7
**Gender (%)**		
Female	63.2	63.1
Male	36.8	36.9
**Co-morbidity (%)**		
Myocardial infarction	8.6	5.7
CHF	17.6	4.5
COPD	24.1	18.8
Diabetes	32.2	17.2
Kidney disease^a^	27.3	21.4

^a^including chronic kidney disease, nephritis, and renal medullary necrosis

**Fig. 2:**
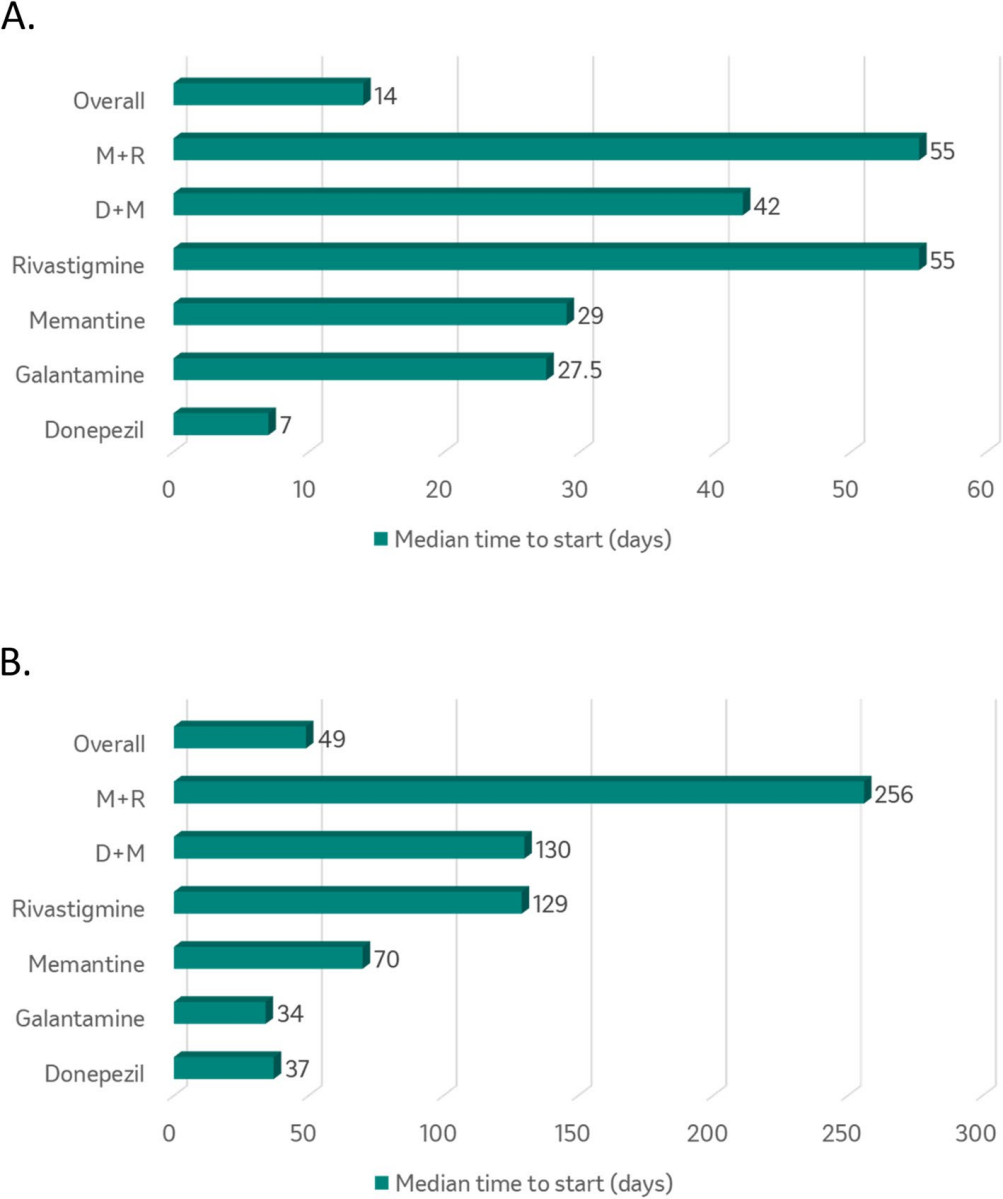
Median time to first symptomatic AD therapy in a major U.S. health plan (**A**) and CPRD (**B**) databases M+R: Memantine + Rivastigamine; D+M: Donepezil + Memantine

**Fig. 3:**
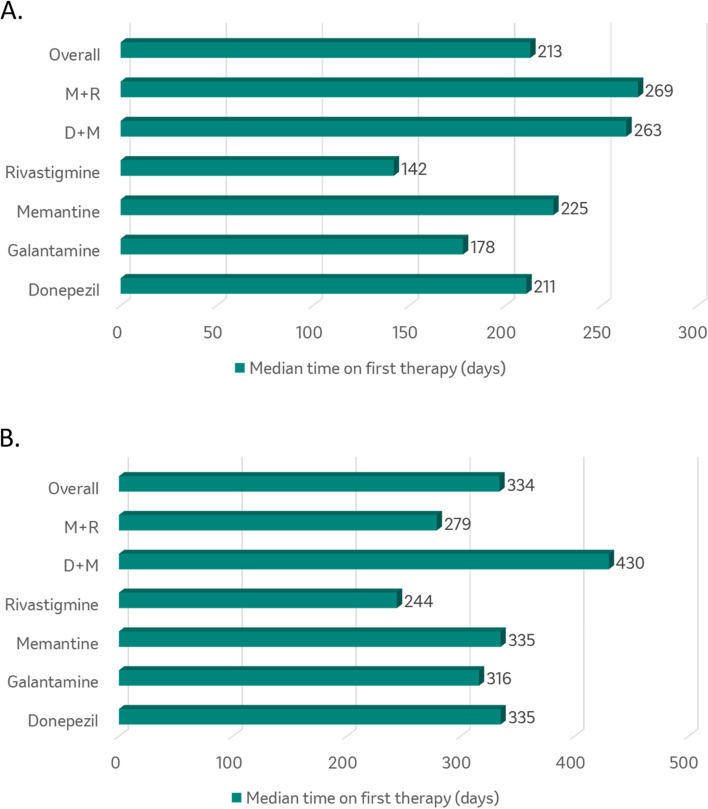
Median time on first symptomatic AD therapy in a major U.S. health plan (**A**) and CPRD (**B**) databases M+R: Memantine + Rivastigamine; D+M: Donepezil + Memantine

The frequency of use of first symptomatic AD treatment for the US cohort are provided in Table 2. Donepezil was used most frequently as the first symptomatic AD therapy (69.4% of patients), followed by memantine monotherapy (19.1%) and donepezil plus memantine combination therapy (6.9%). The overall median time to the start of symptomatic AD therapy was 14 days and ranged from seven days for donepezil to 55 days for rivastigmine (Fig. 2). The overall median time on first symptomatic AD therapy was 213 days (Fig. 3). Only 30% of patients utilized a second symptomatic AD therapy; specifically, donepezil plus memantine combination therapy was used most frequently (54%) followed by memantine monotherapy (24%). Only 3% of patients utilized a third symptomatic AD therapy (Figure S1).

**Table 2:** First symptomatic AD treatment in newly diagnosed AD dementia patients from a major U.S. health plan and CPRD database

	**Major U.S. health plan**	**CPRD**
**N**	7,561^b^	4,470
**Monotherapy**		
Donepezil	5250^a^ (69.4)	2,710 (60.6)
Rivastigmine	252 (3.3)	227 (5.1)
Galantamine	48 (0.6)	221 (4.9)
Memantine	1,447 (19.1)	1,265 (28.3)
**Combination therapy**		
Donepezil + Memantine	518 (6.9)	37 (0.8)
Other	46 (0.7)	10 (0.2)

^a^ < 1% of patients using Donepezil as their first therapy used the 23mg/day dose

^b^Monotherapy is defined as one anti-dementia drug and duration of therapy > 21 days. If monotherapy lasts <  = 21 days, these patients were counted as trial/uncertain category (*n* = 76)

### CPRD GOLD database

As shown in Fig. 1, of the 8,901 newly diagnosed AD dementia patients, 65% received symptomatic treatments during the follow-up period, of whom 77% newly initiated treatment and constituted the final analytic sample. In this sample, there were 4,470 newly diagnosed AD dementia patients without prior treatment history, with 63.1% female and mean age of 81.0 years (SD 7.6 years). Only 2.8% of participants were < 65 years of age. The mean and median follow-up time were 645 and 646 days, respectively. The most frequent co-morbid condition at the index date was chronic renal disease, accounting for 21.4% of the patients (Table 1). Overall the characteristics of newly diagnosed untreated AD dementia patients were similar to those initiating treatment; the untreated cohort tended to be older than the treated cohort (Supplementary Table 2).

The frequency of use of first symptomatic AD treatment for the CPRD cohort is provided in Table 2. Donepezil was used most frequently as the first symptomatic AD therapy (60.6% of patients), followed by memantine monotherapy (28.3%), rivastigmine (5.1%), and galantamine (4.9%). The overall median time to start symptomatic AD therapy was 49 days and ranged from 34 days for galantamine to 256 days for memantine plus rivastigmine (Fig. 2). The overall median time on first symptomatic AD therapy was 334 days (Fig. 3). Only 12% of patients utilized a second symptomatic AD therapy; memantine monotherapy was used most frequently (33%), followed by donepezil plus memantine (26%) and rivastigmine (19%). Only 1% of patients utilized a third symptomatic AD therapy (Figure S1).

## Discussion

Our first key finding in this drug utilization study using recent data is that, despite evidence supporting early initiation and persistent treatment to delay more advanced cognitive and functional deterioration, [21, 22], over a median follow-up of 698 and 645 days, only 63% and 65% of the newly diagnosed AD patients utilized symptomatic treatments for AD dementia, in the US and CPRD databases, respectively. While this study does not explore factors influencing treatment initiation decisions, it underscores the need for further investigation. For instance, this study, as well as previous research, suggest that non-initiators tended to be older and have a higher prevalence of cardiovascular disease (see Supplementary Table S2), [13] potentially due to the rare yet significant heart rate decline associated with cholinesterase inhibitors, contraindicated in patients with unstable or severe cardiac conditions [11].

Donepezil was the most common first-line therapy (> 60%) in this study and had the lowest median time to treatment initiation after AD dementia diagnosis in both the US and UK databases. The findings presented in this study are in line with previous studies reporting that donepezil consistently remained the most commonly used therapy from 2008–2016, accounting for about 69–72% of prevalent anti-dementia drug users [12] and about 68% of treatment initiators in the US [13]. In the context of limited use of an effective disease modifying treatment for AD dementia, donepezil and other cholinesterase inhibitors are used to inhibit the breakdown of acetylcholine, thereby prolonging its action at the synapses [23]. Patients usually start on a low dose and then increase to a higher dose, if the medication is well tolerated. Indeed, the higher dose of donepezil (23mg/day), which has been approved to treat moderate to severe AD dementia, accounted for < 1% of the treatment initiators in this study. Among AD dementia patients using a cholinesterase inhibitor, it was suggested that 40–70% experience temporary symptom improvement for about six to twelve months, yet this effect diminishes and symptoms progressively worsen in the following months [24]. This pattern, and a significant clinical trial dropout due to gastrointestinal side effect and headache, [10] aligns with our observation that the median duration of initial treatment is generally shorter than one year; median time on first therapy was 213 and 334 days in the US and UK databases, respectively.

Rivastigmine is currently the only cholinesterase inhibitor drug available as a patch, and over 70% of rivastigmine users received the drug as a transdermal patch in this study (data not shown). Despite evidence that patches improve adherence and persistence compared to oral administration, [23] the duration of rivastigmine use was the shortest as a first symptomatic AD treatment in both datasets.

Memantine is indicated for moderate to severe AD dementia, and some mild to moderate AD dementia patients take memantine off-label because they cannot tolerate the side effects of the cholinesterase inhibitor drugs. Some AD dementia patients are prescribed the combination treatment of memantine with cholinesterase inhibitor drugs to try and achieve improved effectiveness compared to monotherapy [24]. In this study, memantine was the second most frequently used drug as a first symptomatic treatment. Within second symptomatic AD dementia therapies, memantine monotherapy use and/or usage in combination with donepezil dominated, especially in the US cohort.

Only 30% and 12% of patients, respectively, in the US and CPRD cohorts, progressed to a second symptomatic AD therapy, whereas the others discontinued therapy with < 3% progressing to a third therapy. Long-term treatment and switching from one therapy to another due to intolerable side effects or inadequate response have been shown to be more clinically beneficial than discontinuation, with regards to maintaining function, delaying institutionalization, and managing behavioral issues [25]. Clinicians should clearly communicate practical aspects and anticipated outcomes of symptomatic AD dementia treatment, as it can mitigate but not fully prevent cognitive decline [11].

In addition to side effects and decreased efficacy, medication management in dementia becomes more complex as cognitive function declines, necessitating increased caregiver involvement [26]. However, the extent of patient involvement should not be solely determined by cognitive impairment; various factors, including demographics and psychosocial aspects, influence shared decision-making. Simply excluding patients from this process can negatively impact their quality of life and understanding of their values [27, 28]. Additional research is required to address medication knowledge and communication gaps, emotional stress, financial burdens, and caregiver support, which can impede their roles in adjusting regimens, monitoring safety, communicating with healthcare providers, and participating in decision-making [26, 29]. Collectively, our study highlights the significant research gap concerning adherence and persistence to symptomatic AD treatment within long-term disease management.

Another key finding was the heterogeneity in the pattern of treatment initiation in the US and UK databases. While memantine monotherapy accounted for about 9% higher use as a first symptomatic AD dementia therapy in the CPRD GOLD compared to the US database, the combination use of donepezil and memantine was 6% higher in the US database. Considering the second symptomatic AD dementia therapy, the combination of donepezil and memantine accounted for approximately half of the treatment in the US cohort, while it accounted for only a quarter of the treatment in the CPRD database. In contrast, the use of rivastigmine and galantamine in CPRD were both about three times the corresponding use in the US database. We also observed that in the CPRD database, the time interval after diagnosis to the start of treatment was longer, however, these individuals utilized symptomatic AD dementia medications for a longer period of time than patients in the US cohort.

Furthermore, in the UK most of the newly diagnosed AD dementia patients did not have a record of the use of medications in the pre-diagnosis period (77%) whereas in the US this proportion was much smaller (34%). This suggests that patients are potentially more often being prescribed medications in the US than in the UK before receiving a definitive diagnosis of AD or as part of their diagnostic evaluation. This finding aligns with NICE recommendations that require a consultation with a specialized clinician before prescribing symptomatic AD medications whereas APA guidelines state the cholinesterase inhibitors should be offered to patients with mild to moderate AD dementia [16, 17]. Alternatively, given the limited medical history available in US commercial claims databases, compared with the comprehensive capture of medical history within the UK National Health Service, some of the patients considered to be newly diagnosed with AD dementia in our US study sample may have had previously diagnosed AD dementia initially diagnosed more than 1 year before their index date with no medical encounters for AD within the 12 month pre-index date period. These disparate results from the UK and the US highlight the differences in treatment recommendations and healthcare systems within these 2 countries. Future studies are warranted to understand these differences in greater detail, which may also be attributed to severity of AD dementia at diagnosis, as well as medication coverage and reimbursement.

The strengths of this study include the comprehensive characterization of symptomatic AD dementia treatment patterns in two real-world settings with varying practice guidelines and healthcare systems using recent data from two nationally representative databases. Additionally, the longitudinal study design with up to three-years of follow-up captured changes in treatment, including switching and discontinuation. However, a few limitations should be acknowledged. Similar to all real-world studies conducted using healthcare databases containing medical information that was not collected for research purposes, AD dementia is likely underdiagnosed and the underlying dementia etiology might be misclassified. Furthermore, patients with mild cognitive impairment (MCI) due to AD may have been incorrectly captured within our AD dementia patient cohort. There are codes specific to MCI due to AD (e.g. ICD-10-CM codes G31.83 and F09), but it is unclear how often these codes are used and if clinicians might use AD codes to indicate the presence of MCI. Cognitive testing, biomarker, and imaging results were not available to confirm ICD-10-CM code based AD diagnoses in the data sources used in this study. However, this study required AD dementia patients to have a second diagnosis code for AD after their index diagnosis, which increased the specificity of the AD dementia definition and the likelihood that patients with AD dementia were correctly identified. The data on prescriptions are either based upon physician prescription (CPRD) or filled prescriptions (US database), which may not reflect actual utilization of these medications. Furthermore, the CPRD database captures medical care provided by GPs. However, care for chronic conditions and chronic medication usage should be captured within CPRD even if the care is provided by a specialty physician [19].

## Conclusion

In conclusion, in contemporary cohorts of US and UK based individuals with newly diagnosed AD dementia, approximately two thirds utilized approved symptomatic treatment for AD dementia. Time on first therapy was relatively short (< 1 year) and the majority of patients did not initiate a second therapy. There was significant heterogeneity in the pattern of symptomatic AD treatment initiation in the US and UK databases. These results underscore the need for further investigation of the adherence to existing symptomatic AD therapies. Additionally, the unmet need for therapies to alleviate the significant burden of AD dementia exists within the changing landscape of available therapies, most notably the recent approval of disease modifying treatments targeting brain amyloid for early AD cognitive preservation. As the utilization of disease modifying therapies increases, the treatment patterns of symptomatic AD therapies may be impacted and future studies should examine AD treatment patterns in light of all available therapies.

### Supplementary Information

**Additional file 1: Table S1.** READ codes of Alzheimer's disease. **Figure S1.** Proportion of different symptomatic AD therapy use for the 1^st^, 2^nd^, and 3^rd^ symptomatic therapy in a major U.S. health plan (A) and CPRD (B) databases. **Table S2.** Demographic characteristics and co-morbidities in untreated AD patients at index date from a major U.S. health plan database and CPRD.

## Data Availability

The data that support the findings of this study are available from the corresponding author upon reasonable request.
