# Supplementary material for: Patterns of use of symptomatic treatments for Alzheimer's disease dementia (AD)
Source: BMC Neurol. 2023 Nov 9;23:400. doi: 10.1186/s12883-023-03447-5 (PMC10634008; doi:10.1186/s12883-023-03447-5)
Supplement: Supplementary file 1 — Additional file 1: Table S1. READ codes of Alzheimer's disease. Figure S1. Proportion of different symptomatic AD therapy use for the 1st, 2nd, and 3rd symptomatic therapy in a major U.S. health plan (A) and CPRD (B) databases. Table S2. Demographic characteristics and co-morbidities in untreated AD patients at index date from a major U.S. health plan database and CPRD. [file 12883_2023_3447_MOESM1_ESM.docx]

Supplementary Tables and Figures

Table S1. READ codes of Alzheimer's disease:

| Eu00.00 | Dementia in Alzheimer's disease |
| --- | --- |
| Eu00000 | Dementia in Alzheimer's disease with early onset |
| Eu00011 | Presenile dementia, Alzheimer's type |
| Eu00012 | Primary degen dementia, Alzheimer's type, presenile onset |
| Eu00013 | Alzheimer's disease type 2 |
| Eu00100 | Dementia in Alzheimer's disease with late onset |
| Eu00111 | Alzheimer's disease type 1 |
| Eu00112 | Senile dementia, Alzheimer's type |
| Eu00113 | Primary degen dementia of Alzheimer's type, senile onset |
| Eu00200 | Dementia in Alzheimer's dis, atypical or mixed type |
| Eu00z00 | Dementia in Alzheimer's disease, unspecified |
| Eu00z11 | Alzheimer's dementia unspecified |
| F110.00 | Alzheimer's disease |
| F110000 | Alzheimer's disease with early onset |
| F110100 | Alzheimer's disease with late onset |
| Fyu3000 | Other Alzheimer's disease |

A.


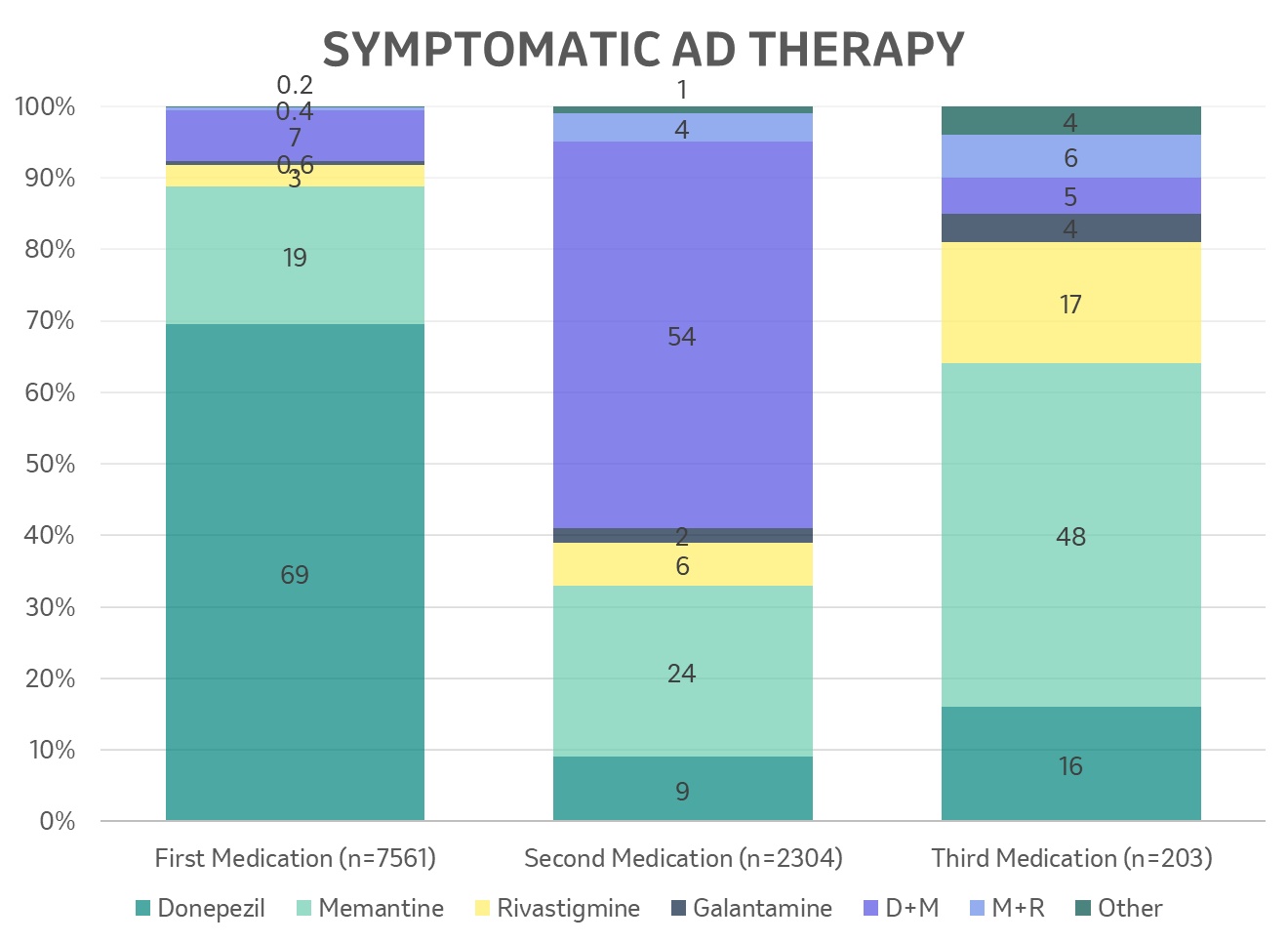


B.


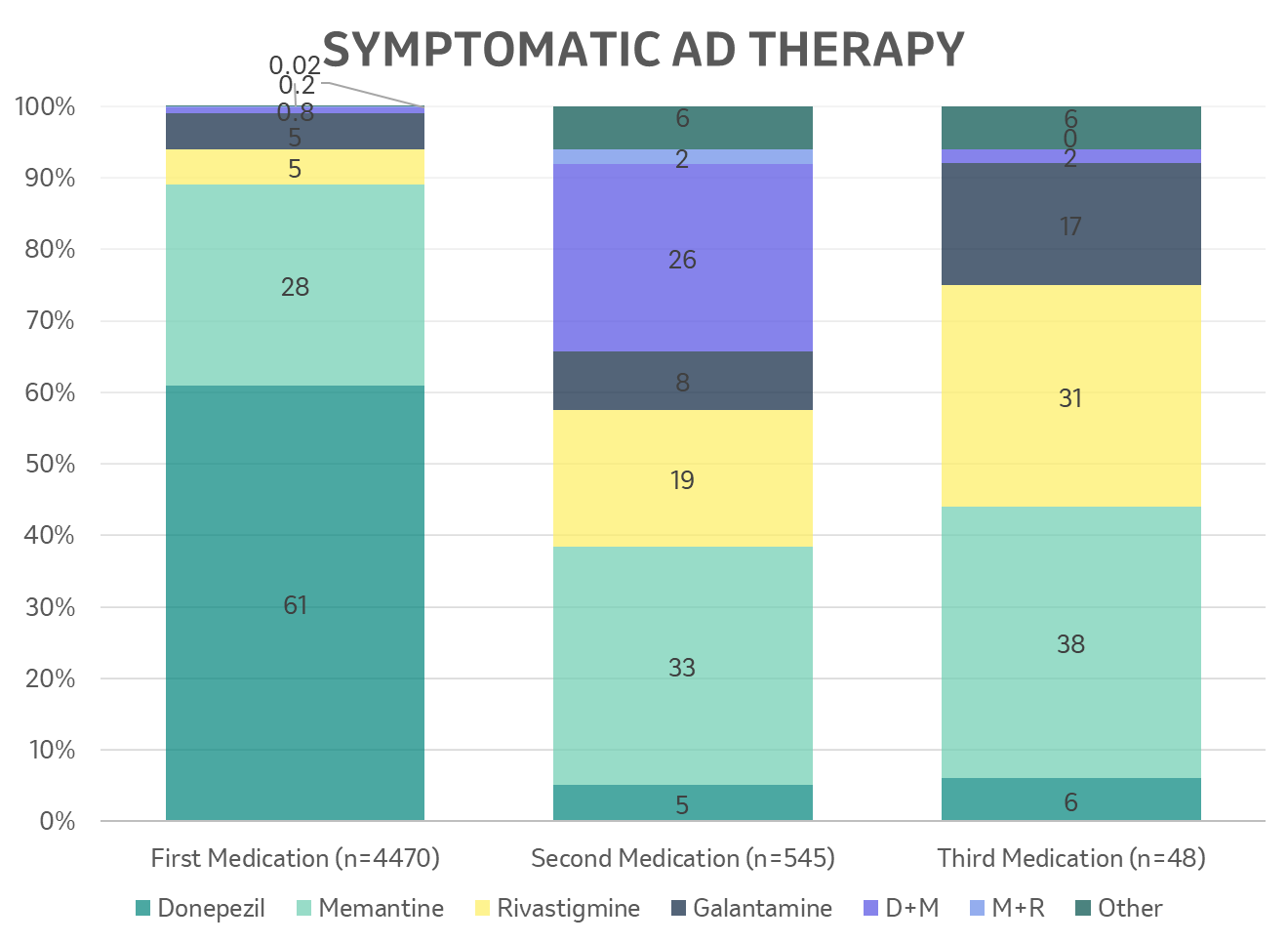


Figure S1. Proportion of different symptomatic AD therapy use for the 1^st^, 2^nd^, and 3^rd^ symptomatic therapy in a major U.S. health plan (A) and CPRD (B) databases.

D+M: Donepezil + Memantine; M+R: Memantine + Rivastigmine

| **Table S2. Demographic characteristics and co-morbidities in untreated AD patients at index date from a major U.S. health plan database and CPRD** | | |
| --- | --- | --- |
| **Characteristics** | **Major U.S. health plan** | **CPRD** |
| **N** | 13,255 | 3,117 |
| **Age (years)** |  |  |
| Mean (std) | 86.6 (14.1) | 83.5 (8.1) |
| **Age Group (%)** |  |  |
| <65 | 1.9 | 2.8 |
| 65-74 | 14.7 | 9.9 |
| 75-84 | 41.7 | 37.9 |
| 85+ | 41.6 | 49.4 |
| **Gender (%)** |  |  |
| Female | 65.8 | 64.6 |
| Male | 34.2 | 35.4 |
| **Co-morbidity (%)** |  |  |
| Myocardial infarction | 9.0 | 6.9 |
| CHF | 22.1 | 6.5 |
| COPD | 24.4 | 19.6 |
| Diabetes | 31.0 | 18.2 |
| Kidney disease* | 27.0 | 23.2 |

**including chronic kidney disease, nephritis, and renal medullary necrosis
